# A temporary deficiency in self‐control: Can heightened motivation overcome this effect?

**DOI:** 10.1111/psyp.12832

**Published:** 2017-01-23

**Authors:** Claire L. Kelly, Trevor J. Crawford, Emma Gowen, Kelly Richardson, Sandra I. Sünram‐Lea

**Affiliations:** ^1^ Department of Psychology Lancaster University Lancaster UK; ^2^ Faculty of Life Sciences University of Manchester Manchester UK

**Keywords:** Self‐control, Self‐awareness, Motivation, Antisaccade task

## Abstract

Self‐control is important for everyday life and involves behavioral regulation. Self‐control requires effort, and when completing two successive self‐control tasks, there is typically a temporary drop in performance in the second task. High self‐reported motivation and being made self‐aware somewhat counteract this effect—with the result that performance in the second task is enhanced. The current study explored the relationship between self‐awareness and motivation on sequential self‐control task performance. Before employing self‐control in an antisaccade task, participants initially applied self‐control in an incongruent Stroop task or completed a control task. After the Stroop task, participants unscrambled sentences that primed self‐awareness (each started with the word “I”) or unscrambled neutral sentences. Motivation was measured after the antisaccade task. Findings revealed that, after exerting self‐control in the incongruent Stroop task, motivation predicted erroneous responses in the antisaccade task for those that unscrambled neutral sentences, and high motivation led to fewer errors. Those primed with self‐awareness were somewhat more motivated overall, but motivation did not significantly predict antisaccade performance. Supporting the resource allocation account, if one was motivated—intrinsically or via the manipulation of self‐awareness—resources were allocated to both tasks leading to the successful completion of two sequential self‐control tasks.

Self‐control is the ability required to override or inhibit an automatic/impulsive response for another, involved in successful behavioral regulation (Baumeister, Heatherton, & Tice, 1994). Self‐control can be applied to many situations, such as suppressing emotions, avoiding distractions at work (e.g., checking social media; Otten et al., 2014). Self‐control is employed regularly every day, and research has estimated that we use self‐control processes approximately 3 to 4 h each day (Hoffman, Baumeister, Foerster, & Vohs, 2012). It is necessary for human social interaction, and there are clear detrimental effects of self‐control failure such as crime, obesity, smoking, and drug problems (Hagger, Wood, Stiff, & Chatzisarantis, 2010).

Despite its importance and regular use, several studies have shown that engaging in self‐control is effortful, and when completing two sequential self‐control tasks, the first task is usually performed well but a temporary deterioration in performance in the second occurs (Hagger et al., 2010). Studies typically employed a sequential self‐control depletion paradigm in which two concurrent self‐control tasks were completed. Frequently employed tasks include the incongruent Stroop (1935) task, a thought suppression task, an attention control video task, and an erasing letters task (Carter, Kofler, Forster, & McCullough, 2015; Hagger et al., 2010). We recently implemented another feasible measure of inhibition—the antisaccade task (Hallett, 1978)—into a sequential self‐control task paradigm (Kelly, Sünram‐Lea & Crawford, 2015).

The strength model/resource depletion theory of self‐control (Baumeister, Vohs, & Tice, 2007) suggested that the temporary deterioration in task performance following self‐control exertion stems from a depletion of limited energy resources. Performing a task necessitating self‐control diminishes those resources, and consequently fewer resources are available, resulting in weakened subsequent self‐control performance.

Glucose was proposed as the relevant physiological energy resource following observation that peripheral glucose levels were significantly reduced following self‐control exertion (Fairclough & Houston, 2004) and that glucose relative to placebo administration restored subsequent self‐control performance following prior exertion (Gailliot et al., 2007). However, recent findings have failed to replicate this, challenging the relationship between glucose availability and self‐control performance (Dang, 2016; Kelly et al., 2015; Kurzban, 2010; Kurzban, Duckworth, Kable, & Myers, 2013; Molden et al., 2012; Sanders, Shirk, Burgin, & Martin, 2012). Although these findings do not necessarily imply that there is no temporary shortage in the energy and more specifically glucose supply centrally, other factors appear to play an important (but not mutually exclusive) role.

For example, level of motivation may be an important moderating factor in self‐control inasmuch as it might ameliorate any self‐control deficiency following prior engagement. That is to say, self‐control is a motivated resource, and motivation determines the effort and time spent on certain tasks/behaviors (Salamore, Correa, Farrar, & Mingote, 2007). Supporting this, administering a monetary incentive for task completion or being told that the tasks were important resulted in an enhanced level of performance in a second self‐control task following initial exertion (Muraven & Slessareva, 2003). Moreover, we previously observed that high levels of self‐reported intrinsic motivation led to enhanced self‐control performance on a second task, whereas those low in motivation showed a deterioration in antisaccade performance after initial self‐control exertion (Kelly et al., 2015).

Increasing levels of self‐awareness appears to have a similar restorative effect on temporary deficiencies in self‐control following prior engagement. Focusing attention on the self can lead to the conscious awareness of the self, a state Duval, Wicklund, and Fine (1972) labeled “objective self‐awareness.” Moreover, it results in a process of self‐evaluation, which consists of comparing the self to a standard of correctness that specifies a state the self ought to have (Duval et al., 1972). Specifically, anything that primes an individual about the self, such as mirrors, hearing one's own voice, or cameras, can increase self‐awareness levels (Stapleton & Smith, 2013; Wicklund, 1979).

Indeed, it has been shown that self‐focused attention has important implications for motivation and self‐regulation (for reviews, see Carver, 2003; Duval & Silvia, 2001; Gibbons, 1990; Silvia & Duval, 2001). For example, previous research demonstrated a positive relationship between self‐focused attention and self‐control. Employing a sequential two‐task depletion paradigm, Alberts, Martijn, and De Vries (2011) used a scrambled sentence task (SST) to induce self‐awareness by priming participants with sentences connected to the self that began with the word “I.” This was administered after the first self‐control task—an auditory suppression task—and before a second self‐control task, which measured perseverance level in a handgrip squeezing task. Those presented with neutral primes showed a temporary deterioration in self‐control performance in the handgrip task and persevered for less time; however, inducing self‐awareness counteracted this.

The finding that motivation and self‐awareness moderate self‐control performance support Beedie and Lane's (2012) resource allocation account. This posits that a temporary deficiency in self‐control is reflective of a reluctance to allocate resources to a task because it is not a personal priority (i.e., considered important and/or interesting). Consequently, the response trajectory of a temporary deficiency in self‐control performance following prior exertion reflects a person's low level of motivation, one unwilling to invest resources (Baumeister, 2014). Applying this to the self‐awareness findings, making an individual more self‐aware arguably might prompt them to improve their performance and motivate them to allocate resources to a second task despite initial exertion.

Alternative models also explain self‐control performance deterioration from a motivational perspective (Inzlicht & Marcora, 2016). Baumeister's amendment to the original resource model suggested that resources are still somewhat diminished during self‐control exertion but, if motivated, any remaining resources are allocated to the subsequent task (Baumeister, 2014; Inzlicht & Schmeichel, in press). The shifting priorities account (Inzlicht & Schmeichel, 2012; Inzlicht, Schmeichel, & Macrae, 2014) suggests that a motivational attentional shift produces the temporary reduction in self‐control; one changes from completing a compulsory task to wanting to perform enjoyable tasks (Baumeister, 2014; Inzlicht, Legault, & Temper, 2014). The “opportunity cost” model suggests that the motivation for task completion stems from the opportunity cost associated with the task (i.e., perception of effort). Motivation is high when a task is perceived as less effortful (Kurzban, et al., 2013).

The current study aimed to further explore the motivational perspectives on self‐control performance and assessed the relationship between self‐awareness and motivation. We manipulated self‐awareness by administering the SST task (Alberts et al., 2011) between two self‐control tasks. Following our previous methodology (Kelly et al., 2015), an initial Stroop (incongruent vs. congruent) task was paired with an antisaccade task in a sequential two‐task paradigm. The prosaccade task was also administered to assess whether completion of an initial self‐control task adversely affected subsequent self‐control performance only or whether the observed effects were extended more generically to other saccade tasks. Secondly, we measured self‐reported levels of motivation using the intrinsic motivation inventory (IMI; McAuley, Duncan, & Tammen, 1989). Based on Alberts et al.'s (2011) findings, we hypothesized that heightening self‐awareness levels would counteract the temporary deficiency in self‐control performance in the antisaccade task following incongruent Stroop task completion. Further, drawing on our recent findings (Kelly et al., 2015), we predicted that high motivation would counteract such temporary decline and lead to sustained antisaccade performance. The relationship between the effects of self‐awareness and motivation on self‐control performance were examined to observe whether priming high self‐awareness would be an intervention that would increase motivation and subsequently attenuate any self‐control deficiency in performance.

## Method

### Participants

We initially tested 61 participants but removed one participant due to the high rate of erroneous responses made in the antisaccade task (89.29%), which indicated that the instructions were not fully understood. On average in the antisaccade task, the error rate for healthy participants is 20% (Hutton, 2008). This resulted in a final sample of 60 healthy young adults (12 male, 48 female) studying at Lancaster University (*M*age = 22.08 years). Before the commencement of the study, a power analysis based on Alberts et al.'s (2011) findings revealed that this sample size was sufficiently highly powered (0.74) according to Cohen's (1988) standards. This study was approved by Lancaster University's Ethics Committee, and written informed consent from all participants was provided according to the Declaration of Helsinki.

### Procedure

Participants attended one testing session, which lasted on average 30 min. Participants were divided into four groups: incongruent Stroop/low self‐awareness, incongruent Stroop/high self‐awareness, congruent Stroop/low self‐awareness, and congruent Stroop/high self‐awareness. Participants first provided written informed consent and then completed either a congruent (control) or incongruent Stroop (which required self‐control) task. The SST was then administered with participants instructed to unscramble 20 sentences to form grammatically coherent statements using five out of the six words available. Participants either received the version that primed self‐awareness or a control (neutral/low self‐awareness) version. Following this, the eye tracking equipment was set up, and participants completed the prosaccade and then antisaccade tasks. This was considered optimal due to evidence of carryover effects between the saccade tasks (Roberts, Hager, & Hare, 1994). After both saccade tasks, participants then completed the IMI, rating how meaningful/important they found the eye movement tasks to complete. At the end of testing, participants were fully debriefed.

### Materials

#### SST

Participants were presented with a list of 20 scrambled sentences and instructed to unscramble each one to form a grammatically correct sentence. The self‐awareness version of the task contained sentences, which when unscrambled began with “I” such as ‘I read books for leisure,” whereas the neutral (low self‐awareness) task contained sentences, which when unscrambled started with different names such as “Catherine reads books for leisure.”

#### IMI

Level of motivation was examined using the 36‐item IMI. Participants made their responses on a 7‐point‐Likert scale, which varied from *not at all true* to *very true*. Example statements that required a response included “I thought this activity was quite enjoyable,” “This activity was fun to do,” “I felt like I had to do this,” and “I think that this activity is useful to me.” An indication of participants’ overall level of motivation was provided by collating and averaging all 36 responses (Li, 2004).

#### Stroop task

This computerized task involved responding to the color (yellow, blue, green, purple, red) of a series of 135 words by pressing relevant keys on a QWERTY keyboard (based on the methodology used by Wallace & Baumeister, 2002). Participants engaged in either a congruent version (control) of the task, in which the ink color and the color words were identical or an incongruent version (depletion), in which they differed (e.g., the word purple was written in green ink). The incongruent task also required one to suppress this instruction when responding to the color *red* and alternatively responding to the written word. After the Stroop task, which was completed in 4 min 30 s, participants answered four questions, which examined different performance outcomes—pleasantness, level of effort exerted, frustration, and tiredness (see Denson, von Hippel, Kemp, & Teo, 2010)—in order to address whether there were differences depending on the two Stroop tasks that were completed.

#### Saccade tasks

Participants completed both a 30‐trial prosaccade task (an eye movement is made toward a presented target) and a 30‐trial antisaccade (Hallett, 1978) task (an automatic prosaccade toward the target is suppressed and an eye movement is directed to the opposite side, away from the target). Participants rested their head on a cushioned chin rest, which was located 57 cm away from a 19″ computer, and the saccade tasks were presented on the screen. An Eyelink 1000 (SR Research: 1,000 Hz, < .5° accuracy) recorded saccadic responses. During both tasks, a fixation cross appeared in the middle of the screen and after an interval of 1,000 ms, the target—a small green dot (.6° diameter)—appeared 8° either to the left or right of the fixation cross. The target and fixation cross both stayed on the screen for 1,000 ms (overlap), and a 1,500‐ms interval preceded the next trial. Target location was randomized and appeared to the left or right of the screen with equal frequency. Calibration and validation procedures before each task were completed, which ensured all recordings were of a good and consistent standard.

### Analysis

All statistical analysis was performed in R (R Core Team, 2015) using the linear mixed effects model package; lme4 (Bates, Maechler, Bolker, & Walker, 2014). For this analysis, as participants completed a series of trials, we included a random effect for participant, to account for individual variation (Winter, 2013). A 2 (Self‐Control condition: self‐control/depletion vs. control) × 2 (Self‐Awareness manipulation: high vs. low/neutral) × 2 (Saccade Task: prosaccade and antisaccade) mixed factorial design with repeated measures on the third factor (saccade task) was conducted. We measured saccade performance in the eye movement tasks based on two specific parameters: saccade latency (response speed) for correct responses and the rate of erroneous responses (for the antisaccade task only). Saccade response speed was calculated using the period between the target onset and the start of the first saccade, with amplitudes of 2° degrees or more. Responses of less than 80 ms and over 500 ms were classified as anticipatory or late saccades, respectively, and removed from the analysis. For the number of errors committed in the antisaccade task, the total number of errors (incorrect saccades made toward rather than away from the target) was obtained relative to the number of correct saccadic responses directed away from the target.

#### Response speed (latency)

We performed a linear mixed effects analysis to examine whether self‐control condition, self‐awareness manipulation, and/or motivation influenced saccadic response speed. Initially, we fitted a null model, which included participant as a random effect. We only had one item (green dot) and thus did not include item as a random effect. We then ran through a series of models, adding task type (prosaccade and antisaccade), self‐control condition (depletion vs. control), and self‐awareness (high vs. neutral/control) as fixed effects, along with motivation as a covariate. We compared models with fixed effects and also those with interactions between the fixed effects using the likelihood ratio test.

#### Correct versus erroneous antisaccade responses

For correct compared to erroneous antisaccade responses, we performed a generalized linear mixed effects analysis. Specifically, we ran through a series of separate models treating participants as random effects and both self‐control condition (depletion vs. control) and self‐awareness condition (high vs. low/neutral) as fixed effects. Self‐reported motivation was then added as a covariate to the models to assess whether differences in motivation significantly predicted the rate of errors compared to correct antisaccade responses.

## Results

### Self‐Reported Performance Differences Based on the Initial Task Completed (Manipulation Check)

We conducted general linear modeling analysis to assess whether self‐reported ratings of task pleasantness, tiredness, frustration, and effort expended differed significantly depending on the initial Stroop task (congruent/control vs. incongruent/depletion) completed. This revealed no significant differences in task pleasantness, *F*(1,58) = 0.20, *p* = .66, or ratings of tiredness, *F*(1,58) = 1.98 × 10^−29^, *p* = 1.00, between the two versions. However, there was a significant effect of frustration, *F*(1,58) = 10.72, *p* < .001; the incongruent (vs. congruent) Stroop task was reported to be more frustrating to complete, β = −1.40, *SE* = 0.43, *t* = −3.28, *p* < .001, than the congruent Stroop task. There was also a significant effect of effort, *F*(1,58) = 30.44, *p* < .001; the congruent was rated as requiring less effort than the incongruent Stroop task, β = −2.20, *SE* = 0.40, *t* = −5.52, *p* < .001.

#### Accuracy

The incongruent version of the Stroop task, which required self‐control, was performed with less accuracy (*M* = 89.57, *SD* = 11.88) than the congruent (control) version (*M* = 99.71, *SD* = .55); specifically those completing the congruent version performed with on average 13.14% greater accuracy, β = 13.14, *SE* = 2.17, *t* = 6.05, *p* < .001.

### Saccade Performance

#### Response speed (latency)

Comparing the null model to a model that also included task as a fixed effect revealed task to be a significant predictor of saccade response speed, χ(1)^2^ = 999.78, *p* < .001; the prosaccade task was performed 60.48 ms ± 1.77 (*SE*s) faster than the antisaccade task. Adding self‐control condition as a fixed effect to the model did not improve the model fit, nor did including self‐awareness condition and motivation and their interactions (*p* > .05). Results showed that participants were faster to perform the prosaccade compared to antisaccade task. The effects of self‐control condition and self‐awareness were not significant. Further, self‐reported levels of motivation did not significantly predict response speed in either task.

#### Correct versus erroneous antisaccade responses

Firstly, fitting a model with self‐control condition (depletion vs. control) as a fixed effect and participants as random effects showed self‐control condition to be not a significant predictor of correct antisaccade responses; those that engaged in the initial depletion (incongruent Stroop) task (*M* = 17.91, *SD* = 15.25) committed a similar rate of errors to those that completed the control (congruent Stroop) task (*M* = 18.03, *SD* = 14.19) β = −0.07, *SE* = 0.28, *Z* = −0.24, *p* = .81. Secondly, we added self‐awareness to the model, which revealed this to be not a significant predictor of responses; those primed with self‐awareness (*M* = 19.24, *SD* = 15.78) produced a comparative rate of errors to those primed with neutral words (*M* = 16.92, *SD* = 13.73) β = 0.17, *SE* = 0.39, *Z* = −0.43, *p* = .67. There was also no significant Self‐Awareness × Initial Condition interaction (*p* = .74). Adding self‐reported levels of motivation produced no significant effect of motivation, β = 0.03, *SE* = 0.50, *Z* = 0.07, *p* = .95, nor was there a significant Initial Condition × Motivation interaction (*p* = .14).

**Figure 1:**
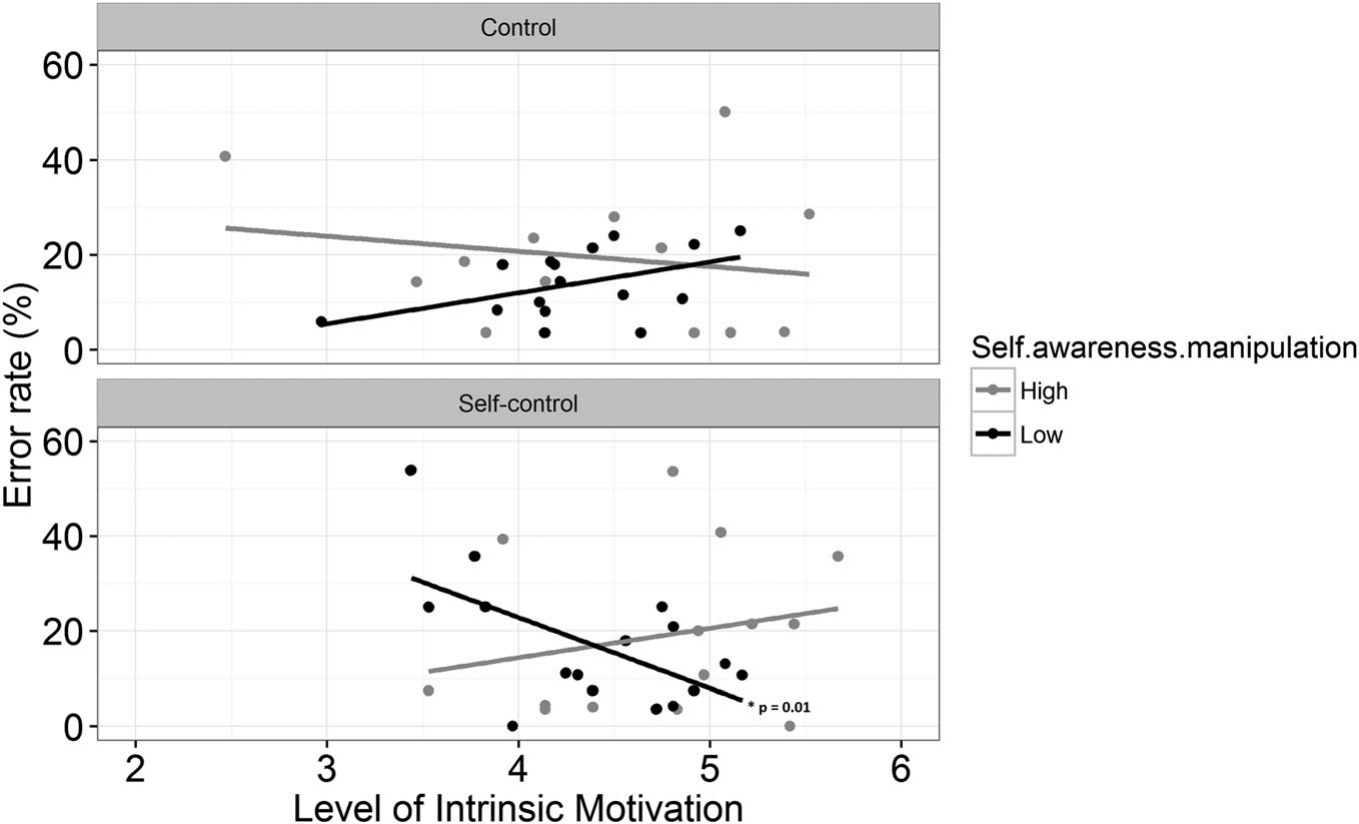
The relationship between motivation, self‐awareness manipulation, and self‐control condition for the proportion of erroneous antisaccade responses.

However, a significant three‐way Self‐Control × Self‐Awareness × Motivation interaction on rate of erroneous responses (see Figure 1) was observed, β = 1.86, *SE* = 0.88, *Z* = 2.11, *p* = .03. Examining this interaction further and splitting by self‐control condition, for participants that completed the incongruent Stroop task (self‐control task), a negative relationship between erroneous responses and motivation was observed, β = −0.96, *SE* = 0. 48, *Z* = −1.99, *p* = .04, indicating that when motivation was high, fewer erroneous responses were made in the antisaccade task. Although self‐awareness alone did not predict erroneous responses in the antisaccade task (*p* > .05), there was a significant Motivation × Self‐Awareness interaction, β = 1.58, *SE* = 0.68, *Z* = 2.34, *p* = .02. Those that had previously applied self‐control (in the incongruent Stroop task) and received the self‐awareness primes performed a similar rate of antisaccade errors regardless of their level of motivation to complete the antisaccade task, β = 0.66, *SE* = 0.58, *Z* = 1.13, *p* = .26. For participants that completed the incongruent Stroop task and were not self‐primed, level of motivation predicted erroneous relative to correct antisaccade responses; those high in motivation produced fewer erroneous responses than those low in motivation, β = −0.98, *SE* = 0.37, *Z* = −2.77, *p* = .01 (see Figure 1). These findings were not extended to the control group (i.e., those participants that first completed the congruent Stroop task).

## Discussion

The current study explored whether the temporary deficiency in performance that is typically observed in the second of two sequential self‐control tasks can be overcome by high motivation and increased self‐awareness. According to the resource depletion theory (Baumeister et al., 2007), the reduction in performance consistently noted in a second of two sequential self‐control tasks stems from self‐control being an effortful process that relies on the availability of a limited energy resource, which reduces through exertion. Based on a previous methodological design (e.g., Kelly et al., 2015), we administered either a congruent (control) or incongruent Stroop task to participants followed by the prosaccade and antisaccade eye movement tasks. However, before the saccade tasks, we manipulated self‐awareness by administering a SST. Self‐reported levels of motivation were also measured using the IMI after the saccade tasks were completed.

The findings revealed that performing an initial self‐control task per se did not predict subsequent self‐control performance. The current data suggest a complex relationship between self‐control exertion, priming of self‐awareness, and level of motivation for correct, compared to erroneous, antisaccade responses. Level of motivation only predicted antisaccade performance when participants were not primed on self‐awareness; those low in motivation committed more erroneous responses than those high in motivation. Thus, only participants with low motivation to perform the second self‐control task showed the typical self‐control depletion effect consistent with the self‐control literature (Hagger et al., 2010), that is, a temporary deficiency in self‐control ability in the second task following prior exertion. When participants were primed on self‐awareness, motivation did not predict subsequent self‐control performance. This opens up the possibility that priming self‐awareness led to an increase in motivation, which in turn counteracted any temporary deficiency in self‐control. These findings are in line with previous research that demonstrated (a) differences in subsequent self‐control performance following the initial exertion of self‐control based on motivation, and (b) no difference in self‐control performance for individuals who were exposed to an explicit manipulation of motivation (Alberts et al., 2011; Kelly et al., 2015; Muraven & Slessarva, 2003). According to Wicklund (1979), raising self‐awareness increases motivation, as the individual is made aware of their performance level, which subsequently increases the motivation to perform a task well.

The findings suggest that an individual who is motivated to complete a task—either through manipulation of self‐awareness or intrinsic high levels of motivation—will successfully engage in a subsequent task of self‐control despite earlier self‐control exertion. This supports growing evidence that one's level of motivation rather than limited resource capacity influences changes in self‐control performance over time (Molden, 2013).

Although the findings are consistent with a motivational account of self‐control, the question arises to what extent motivational factors can compensate for limited resources (Alberts et al., 2011). According to the resource allocation theory (Beedie & Lane, 2012), resources (i.e., glucose) will be assigned based on one's intrinsic level of motivation to complete that task. However, it is as yet unclear which underlying mechanisms determine this allocation of additional energy resources. Specifically, understanding the neurochemical mechanisms behind these findings is needed (Legault & Inzlicht, 2013).

High levels of motivation could trigger an arousal/activation response resulting in energy in the form of glucose to be directed to specific brain areas for successful task completion. Specifically, being motivated to perform a task may have led to activation of the sympathetic adrenal medulla axis, which results in the release of adrenaline (epinephrine) from the adrenal medulla and leads to increase in blood glucose levels. This is in line with recent research, which showed that increasing motivation led to an increase and/or maintenance of blood glucose levels associated with maintenance of performance levels during the second self‐control task. This suggests that being motivated allows allocation of energetic resources to a task, which in turn prevents performance decrements (Kazén, Kuhl, & Leicht, 2015).

Another potential underlying mechanism that might mediate maintenance of performance levels is dynamic change in dopamine activity. Dopamine activity has been associated with a number of psychological processes including motivation. Potts, Martin, Burton, and Montague (2006) have suggested that allocation of resources to limited‐capacity systems might be regulated by dopaminergic reward system input. In the current context, increased dopaminergic activity could be linked with high motivation and the subsequent allocation of energetic and/or cognitive resources to a task. This is supported by recent conceptualizations of dopamine, which suggest the involvement of dopamine beyond solely reward processing (Salamone & Correa, 2012). In particular, the role of dopamine in the nucleus accumbens is considered to be more wide ranging and linked to the engagement of effort and decision making (Salamone et al., 2007).

More specifically, it has been argued that dopamine controls the amount of energy one expends in achieving a goal, particularly when it is considered valuable and important (Salamone et al., 2007). When dopamine levels are higher, one is more engaged in an activity and injects more resources into its completion (Beeler, Frazier, & Zhuang, 2012). For example, Treadway et al. (2012) observed that lower levels of dopamine led one to favor less effortful tasks, whereas enhanced dopamine levels made one willing to expend effort for a reward. In addition, an inverted U‐shaped relationship has been observed between dopamine level and sequential self‐control performance (Dang, Xiao, Liu, Jiang, & Mao, 2016). Participants with “medium” dopamine levels—as measured by eyeblink rate, which is considered a valid measure of dopamine levels (Karson, 1983)—performed well, that is, less erroneously in a second task of self‐control (the antisaccade task) despite initial exertion in a Stroop task compared to those with higher or lower levels.

However, more research is needed to elucidate the role of dopaminergic systems in the complex relationship between self‐control, motivation, and resource allocation,

Consequently, based on the existing evidence, the findings support Beedie and Lane's (2012) resource allocation account that being motivated resulted in resources being allocated to task. Although Baumeister's (2014) amended resource theory accounts for the moderating effect of motivation, it still posits that resources are depleted following self‐control exertion, and as more recent research findings have failed to observe this (Kelly et al., 2015; Molden et al., 2012; Sanders et al., 2012), an account of targeted resource allocation (Beedie & Lane, 2012) seems more appropriate. It is difficult to refute a resource perspective fully, specifically given the evidence on the resource accounts and also given that glucose is an essential energy resource for the brain and vital for cognition. Thus, it seems plausible that glucose is required for self‐control, albeit other factors are likely to moderate this relationship.

Interestingly in the current study, performance differences were only observed for correct compared to erroneous responses and not for response speed in the antisaccade task. As expected, prosaccade responses were significantly faster than antisaccade responses; however, neither self‐awareness nor motivation directly influenced response speed. This replicates our previous study (Kelly et al., 2015), which only observed performance differences based on motivation level for errors performed. This implies a more direct motivational effect for erroneous compared to correct antisaccade responses, which were not influenced by the effects on response speed. As a result, the evidence more strongly supports the observation that being highly motivated counteracts the effects of self‐control deficiency following prior exertion.

Although we replicated Alberts et al.'s (2011) design with the implementation of a SST to induce self‐awareness, it would be interesting if further research expanded these methods by directly manipulating self‐awareness possibly with a mirror, for example, to further assess the link between self‐awareness and self‐control. Moreover, it would also be beneficial to build on the findings of the relationship between self‐reported motivation and self‐control by further manipulating levels of motivation to assess in more detail whether motivation has an ameliorating effect on self‐control deficiency in a similar way.

### Conclusions

This study investigated the effect of self‐awareness and motivation on self‐control performance over time and observed whether a temporary deficiency in performance in the second task following prior exertion could be restored. The findings revealed that, following the exertion of self‐control, self‐reported levels of motivation significantly predicted the rate of erroneous responses for those not exposed to the self‐awareness primes. When self‐awareness was induced, there were no differences in antisaccade responses based on motivation level. This arguably supports a motivation resource account; following the application of self‐control, if one is motivated to perform a second self‐control task—stemming from self‐awareness resulting in one wanting to perform well or, if this is not induced, based on how interesting and/or enjoyable the task or tasks were perceived to be—this has a restorative effect on a temporary deficiency in self‐control ability, leading one to allocate resources and perform the second task well. This supports the idea of self‐control performance based on more targeted allocation of resources rather than depletion and shows that interventions targeted at motivation can help overcome the effect of impaired self‐control performance following prior exertion.
